# Induction of Labor and Risk of Postpartum Hemorrhage in Low Risk Parturients

**DOI:** 10.1371/journal.pone.0054858

**Published:** 2013-01-25

**Authors:** Imane Khireddine, Camille Le Ray, Corinne Dupont, René-Charles Rudigoz, Marie-Hélène Bouvier-Colle, Catherine Deneux-Tharaux

**Affiliations:** 1 INSERM U953 Epidemiological Research Unit on Perinatal Health and Women’s and Children’s Health, Université Pierre et Marie Curie Paris6, Paris, France; 2 Maternité de Port-Royal, Groupe hospitalier Cochin-Broca –Hôtel Dieu, assistance publique Hôpitaux de Paris, Université Paris Descartes, Sorbonne Paris Cité, Paris, France; 3 Aurore Perinatal network, Hôpital de la Croix Rousse, Hospices Civils de Lyon, EA 4129 Université Lyon 1, Lyon, France; Baylor College of Medicine, United States of America

## Abstract

**Objective:**

Labor induction is an increasingly common procedure, even among women at low risk, although evidence to assess its risks remains sparse. Our objective was to assess the association between induction of labor and postpartum hemorrhage (PPH) in low-risk parturients, globally and according to its indications and methods.

**Method:**

Population-based case-control study of low-risk women who gave birth in 106 French maternity units between December 2004 and November 2006, including 4450 women with PPH, 1125 of them severe, and 1744 controls. Indications for labor induction were standard or non-standard, according to national guidelines. Induction methods were oxytocin or prostaglandins. Multilevel multivariable logistic regression modelling was used to test the independent association between induction and PPH, quantified as odds ratios.

**Results:**

After adjustment for all potential confounders, labor induction was associated with a significantly higher risk of PPH (adjusted odds ratio, AOR1.22, 95%CI 1.04–1.42). This excess risk was found for induction with both oxytocin (AOR 1.52, 95%CI 1.19–1.93 for all and 1.57, 95%CI 1.11–2.20 for severe PPH) and prostaglandins (AOR 1.21, 95%CI 0.97–1.51 for all and 1.42, 95%CI 1.04–1.94 for severe PPH). Standard indicated induction was significantly associated with PPH (AOR1.28, 95%CI 1.06–1.55) while no significant association was found for non-standard indicated inductions.

**Conclusion:**

Even in low risk women, induction of labor, regardless of the method used, is associated with a higher risk of PPH than spontaneous labor. However, there was no excess risk of PPH in women who underwent induction of labor for non-standard indications. This raises the hypothesis that the higher risk of PPH associated with labor induction may be limited to unfavorable obstetrical situations.

## Introduction

In most developed countries, induction of labor is an increasingly common obstetric procedure [1]–[3]. It has been medically indicated for decades in women at high risk to prevent the risks associated with the prolongation of pregnancy and national guidelines listing these indications have been established [4]–[6]. In these situations, it has been associated with improved maternal and neonatal health outcomes [7]–[10]. The issue is different for low-risk women, most of whom are expected to start labor spontaneously, without needing medical induction. Several reports have shown, however, that labor induction has also become a common procedure in this group and that its use has been extended to non-standard indications or even reasons of convenience [11]–[16]. This trend is of particular concern because evidence regarding the potential risks associated with induction is inconclusive, so that the risk-benefit ratio is difficult to evaluate, especially in the low-risk population.

Postpartum hemorrhage (PPH), one of the leading causes of maternal mortality and severe morbidity [17], [18], is one possible risk of induced labor. Several studies of PPH risk factors reported a significant association between labor induction and hemorrhage [19], [20]. However, because these analyses did not take the women’s obstetric history completely into account, the possibility that the underlying indication for induction might explain the excess number of PPHs rather than the procedure itself (indication bias) cannot be ruled out. Characterization of the methods and indications for induction appears necessary for a better understanding of this association. Other observational studies [19], [21]–[23] and randomized controlled trials [7] have compared elective induction to spontaneous onset of labor in low risk parturients and included PPH as a secondary outcome. Although most of them found no excess risk of PPH in the induction group, they generally lacked the power to detect a difference between the two groups for this outcome.

Our objective was to study the association between induction of labor and PPH in women at low risk, according to its methods and indications.

## Methods

### We conducted a population-based cohort-nested case-control study

The study population included women selected from the Pithagore6 trial population [24]. This cluster-randomized controlled trial was conducted between December 2004 and November 2006 in 106 French maternity units of three French regions representing 17% of all French maternity units and covering 20% of deliveries nationwide. Its main objective was to evaluate a multifaceted intervention for reducing the rate of severe PPH. No significant difference in the rate of severe PPH was found between the group of units who received the intervention and the reference group of units where no intervention was conducted (see reference for full description of the original study [24].

PPH was clinically defined as an estimated blood loss greater than 500 mL within the first 24 hours after the birth. Birth attendants in each unit prospectively identified all deliveries with PPH and reported them to the research team. In addition, a research assistant reviewed the delivery suite logbook of each unit and checked any available computerized patient charts. For every delivery with a mention of PPH, the patient’s obstetrics file was further checked to verify the PPH diagnosis.

During the data collection period, 6660 cases of PPH occurred among 146,781 deliveries in the 106 maternity units for a total incidence of 4.5% of deliveries. During the same period, a representative sample of women with deliveries without PPH in the same units was recruited by a random selection of 1/60 of deliveries (ratio based on an estimated incidence of severe PPH of 1/60), to serve as controls in a variety of studies such as this one.

To meet the objectives of this study, we first selected from the Pithagore6 population a population of low-risk parturients defined as women who gave birth to a live singleton fetus in cephalic presentation at a gestational age ≥37 weeks. Women were excluded if they had a condition likely to introduce an indication or confounding bias in the association between induction of labor and PPH, such as coagulopathy or other chronic disease before pregnancy, pregnancy-induced disease (including gestational diabetes, pregnancy-induced hypertension, preeclampsia, placenta abruptio, HELLP syndrome, placenta praevia, chorioamniotitis), antiplatelet and anticoagulant drugs taken during pregnancy, fetus with congenital malformation, previous cesarean delivery or uterine scar. Lastly, as the exposure of interest was the induction of labor, women who had a cesarean delivery before onset of labor were also excluded.

For this case-control analysis, we defined two groups of cases based on the severity of PPH. The first group of cases included all women with PPH from the selected low-risk population. The second group of cases included women with severe PPH, defined by a peripartum decrease in Hb ≥ 4 g/dL (considered equivalent to a blood loss ≥ 1000 mL) or red blood cell (RBC) transfusion ≥ 2 units. Prepartum Hb was collected as part of routine prenatal care during the last weeks of pregnancy; postpartum Hb was the lowest Hb level measured during the 3 days after delivery.

**Figure 1:**
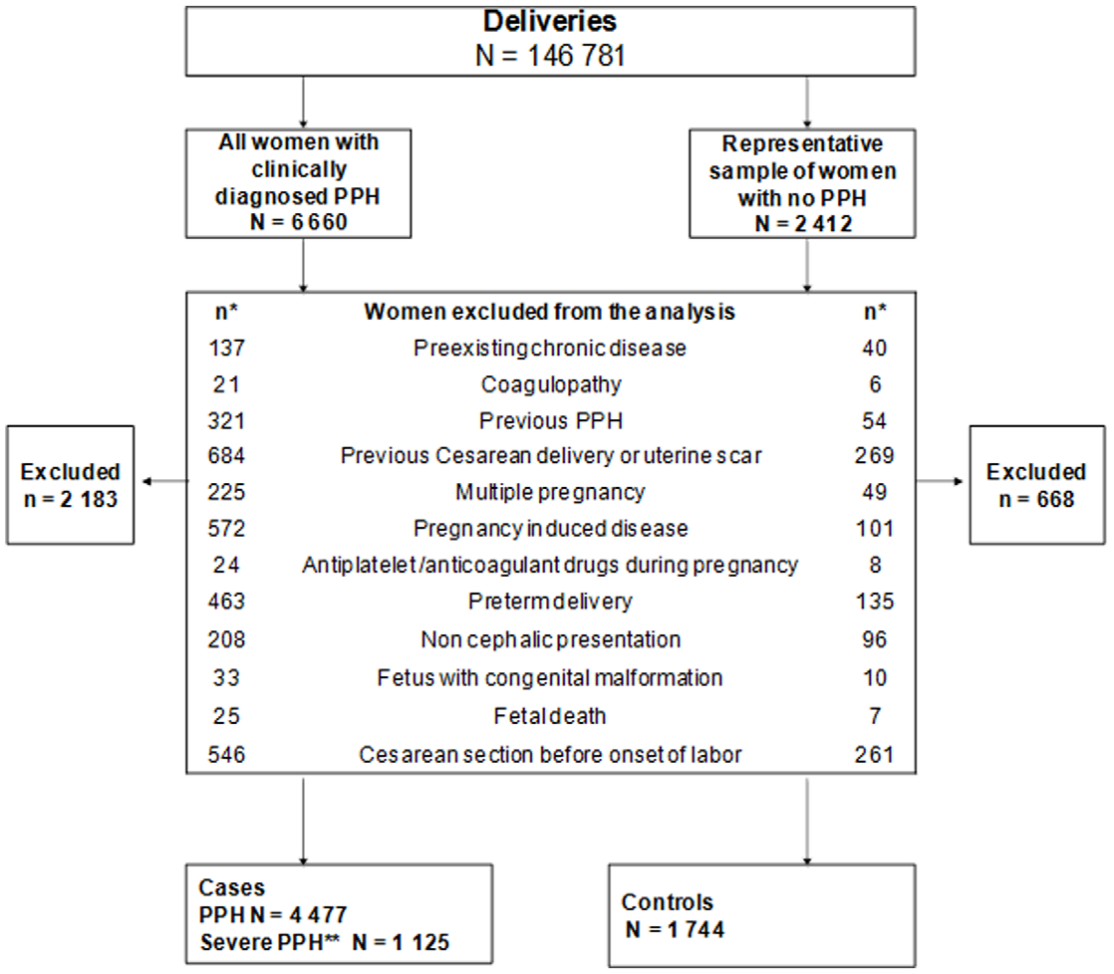
Selection of the study population.

Women without PPH randomly selected for the control population and who met the criteria for low risk served as controls in this study. Finally the study included 4477 women with PPH, 1125 of whom had severe PPH, and 1745 controls. Figure 1 shows the process of selection of the study population.

Characteristics of the patient, pregnancy, labor and delivery were collected from the chart of every delivery. Those included the type of onset of labor (spontaneous or induced) and, if labor was induced, the indication and method of induction as reported in the medical files by the midwife or obstetrician.

We characterized induction of labor with two different variables, one describing its indication and the other its method. Based on the indication stated by the clinician in the medical files, the first variable categorized the indication for induction as standard or non-standard; standard indications were medically indicated procedures according to the French guidelines [4] and included premature rupture of membranes (PROM), postterm pregnancy (delivery at or after 41 weeks of gestation), fetal compromise (suspected fetal growth restriction, oligohydramnios, or abnormal fetal heart rate), prior fetal death or prior precipitate labor; nonstandard indications included other medical reasons not in the guidelines and inductions for convenience or with no specified indication. The second variable characterizing method of labor onset was in four classes: induction with intravenous oxytocin, induction with cervical ripening with prostaglandins, spontaneous onset with secondary augmentation of labor with intravenous oxytocin, and spontaneous onset without augmentation of labor (reference class); oxytocin and cervical ripening with prostaglandins were the only methods used for labor induction in this population. In the subgroups where oxytocin was administered, the total dose of oxytocin received was reported.

Covariables included maternal age at delivery, body mass index (BMI) at conception, parity, gestational age at delivery, epidural analgesia, duration of the active first phase of labor (i.e between 3 cms and complete cervical dilation) in minutes (categorized using the 50 th, 75 th and 90 th percentiles of its distribution in controls), mode of delivery, episiotomy or perineal tears, birth weight and prophylactic oxytocin in the third stage.

In accordance with the case-control design of the study, the characteristics of labor induction were described in the control group, as this group reflects the population of low-risk parturients.

The bivariate analysis compared the characteristics of cases and controls with χ^2^ or Fisher exact tests. The independent effects of labor induction on the risk of PPH and severe PPH were tested with multivariate logistic regression models. Given the hierarchical structure of our data, we used multilevel logistic regression models with a random intercept for maternity units to take into account the intraclass (or intracluster) correlation for outcomes of women cared for at a given center. Covariables included in these models were risk factors for PPH that appeared to be potential confounders in the bivariate analysis (p<0.1). As post term pregnancy was the main standard indication for induction, the gestational age at delivery was not included in the multivariate analyses to avoid over adjustment. In addition, regression models with severe PPH as the dependent variable were also adjusted for the proportion of women with PPH who had a postpartum Hb measurement in each unit (level 2 covariable) because this proportion was heterogeneous between units (from 74% to 99%).

Clinically relevant interactions between induction of labor and covariables (parity and mode of delivery) were tested and none was significant. The rate of missing values was less than 3% among both cases and controls for all variables, except BMI and duration of active phase of labor for which we created a specific missing value indicator variable for the regression analyses.

Because of the specificities of labor and delivery among primiparas, we performed the same analysis in this subgroup of low-risk primiparas.

Based on our sample size of 4477 women with PPH (and 1125 with severe PPH) and 1744 controls, and an exposure prevalence of 5% among controls, we estimated that the power of the study would be 100% to detect an OR of 2 and 95% to detect an OR of 1.5 for all PPH, and 100% to detect an OR of 2 and 75% to detect an OR of 1.5 for severe PPH. Analyses were performed with Stata v.11 software (Stata Corporation, college station TX, USA).

Individual consent was not needed in this study. Collective information about the study was provided in all maternity units and women had the possibility to deny the use of data from their medical files. The principle of non-opposition was applied. The Sud Est III Institutional Review Board and the French Data Protection Agency (CNIL) provided approval for the study.

## Results

Of the 1744 low-risk women in the control group, labor was induced for 316 (18.1%). Among the latter, the indication was standard for 196 (62.0%) and non-standard for 120 (38.0%) (Table 1). The primary standard indications were post term pregnancy in 150 (76.5%) women, and premature rupture of membranes in 35 (17.8%) women. Non-standard indications were most often convenience inductions or inductions with no specified indication in 81 (67.5%) women (Table 1). The method of induction varied with the indication; in standard indications, the main method used was cervical ripening in 123 (62.8%) women, whereas oxytocin was mainly used for nonstandard inductions in 70 (58.3%) of women (p<0.01 for χ^2^ test).

**Table 1:** Indication for induction of labor among control women (N = 316).

	n	%*	n (%)
**Standard indications for induction**			**196 (62.0)**
Post term pregnancy (term>41wks)	150	76.5	
Premature rupture of membranes	35	17.8	
Abnormal fetal heart rate	18	9.2	
Oligoamnios	8	4.1	
Suspected fetal growth restriction	6	3.1	
Meconial amniotic fluid	5	2.5	
Prior fetal death	2	1.0	
**Non standard indications for induction**			**120 (38.0)**
Convenience or no specified indication	81	67.5	
Suspected fetal macrosomia	11	9.2	
Reported “Post term” pregnancy (but <41 wks)	10	8.3	
Isolated decreased fetal movement	8	6.7	
Placental calcification	4	3.3	
Isolated edema	3	2.5	
Isolated proteinuria	2	1.6	
Isolated hyperuricemia	2	1.6	
Others**	8	6.7	
**Total**			**316 (100)**

All control women with induced labor

* Percentages will not add to 100% because indications are not mutually exclusive

** Include: Isolated hypertension, nausea and vomiting, isolated epigastralgic pain, pruritus, metrorrhagia, hydramnios.

Neither the proportion of women with induced labor nor the indications and methods of induction varied significantly by the characteristics of the maternity units (status - university, other public, or private - and annual number of deliveries) (data not shown).

The bivariate analysis shows that labor was induced more often among women with PPH and severe PPH than among the controls (p<0.01) (Table 2). Cases and controls also differed significantly when considering the indications (p<0.01) and methods of labor induction (p<0.01) (Table 2). The mean total dose of oxytocin received during labor was significantly greater among PPH cases than among the controls 1.52 +/− 0.04 and 0.95 +/− 0.06 UI, p <0.01 for Kruskall Wallis test); and greater among induced women than in women with spontaneous onset of labor, among both cases (3.05 +/− 0.09 and 1.10 +/− 0.03 UI respectively, p<0.01 for Kruskall Wallis test) and controls (2.04 +/− 0.13 and 0.71 +/− 0.13 UI respectively, p<0.01 for Kruskall Wallis test). Other characteristics that were more common among case women were: maternal age<25 years, primiparity, postterm pregnancy, epidural analgesia, prolonged active phase of labor, instrumental vaginal delivery, episiotomy, macrosomia and the absence of prophylactic oxytocin in the third stage of labor (Table 2). After adjustment for maternal, labor and delivery characteristics in the multivariate analysis, induced labor was associated with a significantly increased risk of PPH as compared to spontaneous labor (OR 1.22, 95%CI 1.04–1.42) (Table 3). When labor induction was analyzed according to its indication, compared to spontaneous onset of labor, induction for standard indications was associated with a higher risk of PPH (OR 1.28, 95%CI 1.06–1.55) and of severe PPH (OR 1.33, 95%CI 1.04–1.71), while the associations were not significant for non-standard indications. When labor induction was analyzed according to its method, induced labor with oxytocin was associated with a significantly higher risk of PPH (OR 1.52, 95%CI 1.19–1.93) and severe PPH (OR 1.57, 95%CI 1.11–2.20) compared to women with spontaneous labor without augmentation; induced labor with cervical ripening was also significantly associated with severe PPH (OR 1.42, 95%CI 1.04–1.94); women who had spontaneous onset of labor with administration of oxytocin for labor augmentation had an increased risk of both PPH (OR 1.17, 95%CI 1.00–1.37) and severe PPH (OR 1.35, 95%CI 1.07–1.70) (Table 3).

**Table 2:** Characteristics of women, labor and delivery in women with PPH, severe PPH and in control women.

	Controls	PPH cases	P**	Severe PPH cases	P***
	N = 1744	N = 4477		N = 1125	
	n (%)*	n (%)*		n (%)*	
**Induced labor**	316 (18.1)	964 (21.5)	<.01	245 (21.8)	0.01
**Onset of labor with indication of induction**					
Spontaneous	1428 (81.9)	3513 (78.5)		880 (78.2)	
Standard indication for induction	196 (11.2)	663 (14.8)	<.01	185 (16.4)	<.01
Non-standard indication for induction	120 (6.8)	301 (6.7)		60 (5.3)	
**Onset of labor with method of induction**					
Spontaneous without augmentation	607 (34.9)	1258 (28.2)		274 (24.4)	
Spontaneous with augmentation	818 (47.0)	2247 (50.3)		604 (53.8)	
Induction with cervical ripening	173 (9.9)	521 (11.7)	<.01	148 (13.2)	<.01
Induction with oxytocin	143 (8.2)	440 (9.8)		97 (8.6)	
**Women and pregnancy**					
Maternal age (years)					
<25	268 (15.4)	804 (18.0)		210 (18.7)	
25–34	1183 (67.9)	3015 (67.4)	0.01	767 (68.2)	<.01
≥35	292 (16.7)	653 (14.6)		147 (13.1)	
BMI (kg/m^2^)					
<25	1172 (80.0)	3127 (79.3)		803 (80.9)	
25–29	220 (15.0)	572 (14.5)	0.31	138 (13.9)	0.74
≥30	74 (5.0)	241 (6.2)		52 (5.2)	
Missing data	278 (16)‡	537 (12.0)‡		132 (11.7)‡	
Primiparity	809 (46.4)	2678 (59.8)	<.01	786 (69.9)	<.01
**Labor & delivery characteristics**					
Gestational age (weeks)					
37–38	357 (20.5)	646 (14.4)		149 (13.3)	
39–40	1058 (60.7)	2649 (59.3)	<.01	645 (57.4)	<.01
≥41	328 (18.8)	1175 (26.3)		329 (29.3)	
Epidural analgesia	1297 (74.4)	3568 (79.7)	<.01	878 (78.0)	0.02
Duration of active first phase of labor					
< P50 of controls	833 (49.5)	1514 (35.2)		355 (33.4)	
[P50–P75]	425 (25.3)	1093 (25.4)		238 (22.4)	
[P75–P90]	262 (15.6)	919 (21.4)	<.01	242 (22.8)	<.01
≥P90	161 (9.6)	778 (18.0)		228 (21.4)	
Missing data	62 (3.6)‡	173 (3.9)‡		62 (5.5)‡	
Mode of delivery					
Spontaneous vaginal	1422 (81.5)	3251 (72.6)		697 (62.0)	
Instrumental vaginal	207 (11.9)	955 (21.3)	<.01	321 (28.5)	<.01
Emergency cesarean section	115 (6.6)	271 (6.1)		107 (9.5)	
Episiotomy	551 (31.6)	2027 (45.3)	<.01	611 (54.3)	<.01
Perineal tears	501 (28.7)	1345 (30.0)	0.30	317 (28.2)	0.75
Birth weight (g)					
<3000	350 (20.1)	576 (12.9)		130 (11.5)	
3000–3499	757 (43.4)	1747 (39.1)	0.31	439 (39.0)	<.01
3500–3999	519 (29.8)	1611 (36.0)		418 (37.2)	
≥4000	116 (6.7)	539 (12.0)		138 (12.3)	
Prophylactic oxytocin after birth	1253 (71.9)	2609 (58.3)	<.01	697 (62.0)	<.01

* % of non-missing values

‡ % of all women in the group

** chi2 test comparing PPH cases and controls

*** chi2 test comparing severe PPH cases and controls

**Table 3:** Association between induction of labor and risk of PPH and severe PPH in low-risk women, multivariable analyses* (N = 4477 PPH, 1125 severe PPH and 1744 controls).

	Adj OR**PPH	Adj OR***Severe PPH
**Onset of labor**		
Spontaneous	*Ref*	*Ref*
Induced labor	1.22 [1.04–1.42]	1.20 [0.97–1.48]
**Onset of labor with indication of induction of labor**		
Spontaneous	*Ref*	*Ref*
Standard indication for induction	1.28 [1.06–1.55]	1.33 [1.04–1.71]
Non-standard indication for induction	1.11 [0.89–1.40]	0.96 [0.68–1.36]
**Onset of labor with method of induction of labor**		
Spontaneous without augmentation	*Ref*	*Ref*
Spontaneous with augmentation	1.17 [1.00–1.37]	1.35 [1.07–1.70]
Induction with cervical ripening	1.21 [0.97–1.51]	1.42 [1.04–1.94]
Induction with oxytocin	1.52 [1.19–1.93]	1. 57 [1.11–2.20]

* 6 multileveled logistic regression models with random intercept.

** 3 models adjusted for maternal age, parity, epidural analgesia, duration of active phase of labor, mode of delivery, episiotomy/perineal tears, prophylactic oxytocin after birth and birth weight.

*** 3 models adjusted for maternal age, parity, epidural analgesia, duration of active phase of labor, mode of delivery, episiotomy/perineal tears, prophylactic oxytocin after birth, birth weight and % of PPH with no documented Hb delta (level 2 covariable)

The specific analysis among primiparas showed that induced labor was significantly associated with PPH in this population as well (OR 1.27, 95%CI 1.03–1.58) (Table 4). Associations of PPH and induction according to its indications and methods were similar to those found in the whole population.

**Table 4:** Association between induction of labor and risk of PPH and severe PPH among low-risk primiparas, multivariable analyses*.

	ControlsN = 809n (%)	PPH casesN = 2678n (%)	P value	Adj OR**PPH	Severe PPH casesN = 786n (%)	P value	Adj OR***Severe PPH
**Onset of labor**							
Spontaneous (ref)	657 (81.2)	2076 (77.5)	<.01	*Ref*	612 (77.9)	0.02	*Ref*
Induced labor	152 (18.8)	602 (22.5)		1.27 [1.03–1.58]	174 (22.1)		1.22 [0.93–1.61]
**Indication of induction of labor**							
Spontaneous (ref)	657 (81.2)	2076 (77.5)		*Ref*	612 (77.8)		*Ref*
Standard indication for induction	108 (13.4)	440 (16.4)	<.01	1.35 [1.04–1.74]	142 (18.1)	<.01	1.43 [1.04–1.97]
Non-standard indication for induction	44 (5.4)	162 (6.1)		1.13 [0.80–1.60]	32 (4.1)		0.79 [0.48–1.29]
**Method of induction of labor**							
Spontaneous without augmentation (ref)	196 (24.3)	499 (18.7)		*Ref*	136 (17.3)		*Ref*
Spontaneous with augmentation	460 (56.9)	1571 (58.8)	<.01	1.18 [0.93–1.50]	475 (60.5)	<.01	1.39 [1.01–1.91]
Induction with cervical ripening	97 (12.0)	396 (14.8)		1.46 [1.06–2.00]	123 (15.7)		1.71 [1.13–2.56]
Induction with oxytocin	55 (6.8)	205 (7.7)		1.43 [0.98–2.11]	51 (6.5)		1.35 [0.81–2.24]

* 6 multileveled logistic regression models with random intercept.

** 3 models adjusted for maternal age, parity, epidural analgesia, duration of active phase of labor, mode of delivery, episiotomy/perineal tears, prophylactic oxytocin after birth and birth weight.

*** 3 models adjusted for maternal age, parity, epidural analgesia, duration of active phase of labor, mode of delivery, episiotomy/perineal tears, prophylactic oxytocin after birth, birth weight and % of PPH with no documented Hb delta (level 2 covariable)

## Discussion

We found that induction of labor was independently associated with a 20 % higher risk of PPH and severe PPH in low-risk parturients, regardless of the method of induction used. This excess risk of PPH and severe PPH was significant for standard but not for non-standard indications.

Our study design had several strengths. Although the data were extracted from a cluster-randomized trial, the study was population-based as it covered all maternity units in a given area and consequently all women delivering in this area and more specifically, all women with PPH; the characteristics of maternity units and parturient women were comparable to the national picture on the whole [1], and, in particular, for the characteristics of labor induction [14]. Women with PPH and the control subjects were selected from the same source cohort of deliveries, which decreased the likelihood of selection bias. The study included a large number of women with PPH, which allowed the study of rare exposures, although the power was still limited for the rarest categories. Contrasting with previous studies [19]–[23], [25] the detailed information directly collected from medical files made it possible to classify labor induction into different categories of indications and methods, and not only as a binary variable (spontaneous versus induced labor). Finally, the use of multilevel models was relevant to explore the role of exposures and outcomes that potentially vary between units.

Previous studies exploring PPH risk factors have reported an increased risk associated with labor induction [19], [20]; however, they did not select a low risk population [19], [20] and/or did not adjust for duration of labor—a major confounder—[20], which made it possible that the association they reported actually reflected indication bias and/or residual confounding. Other studies of PPH risk factors have reported no significant impact of labor induction [25] but they were based on retrospective administrative data, whose validity may be limited for exploring etiologic aspects of health outcomes. Our analysis conducted in a low risk population and taking into account all potential confounders provides valuable additional evidence of an association between labor induction and PPH.

Among the primiparas, we found results similar to those found in the total population. Previous studies reported an absence of association between induction and PPH in primiparas with either favorable [23] or unfavorable cervices [22]; however, they were inadequately powered to study such rare outcomes.

Several hypotheses might explain the higher risk of PPH and severe PPH after induction of labor. First, the drugs used to induce labor might have a direct effect on the uterine muscle and could, by causing supra physiological contractions, act as a fatigue factor on the myometrium muscle and thus, lead to postpartum atony and possibly PPH [26]–[28].

In addition, as oxytocin is administered throughout labor in nearly all women with inductions, this higher risk of PPH could also be mediated by the cumulative effect of this drug on the uterine muscle [29]. This would explain our finding that induction is associated with PPH, regardless of the method used. Indeed, several recent studies have reported an increased risk of PPH associated with augmentation of labor, independently of the manner of its onset [30], [31]. Our finding, in this low-risk population, that women with spontaneous onset of labor but subsequent labor augmentation are at higher risk of PPH than women with spontaneous labor and no augmentation provides further support for this hypothesis. The nearly universal use of oxytocin during labor among women with induced labor makes it collinear with our exposure of interest and prevents us from adjusting for this variable to verify whether this intermediary factor completely explains the association between induction and PPH.

Finally, although we adjusted for the duration of the active phase of labor, other unexplored aspects of labor, such as the duration of the latency phase or the dynamics of labor, might be specific in women with induced labor and act as confounders or intermediary factors in the relation between induction and PPH.

This latter hypothesis may explain why the increased risk of PPH associated with labor induction appears limited to situations where this procedure is performed for standard indications. Although Bishop scores were not available in this study, most of the standard inductions were performed by cervical ripening, in contrast to non standard indications, and thus suggests that these women are more likely to have an unfavorable cervix. Prolonged active phase of labor—an independent risk factor for postpartum hemorrhage [32], [33]—may be more common in women with unfavorable cervix, and explain the association between standard induction and PPH; however, the fact that this association remains significant when we adjusted for the duration of the active phase shows that the effect of induction on the risk of PPH is not fully mediated by a longer duration of the active phase of labor. Other specificities of labor—such as long latency phase or need for labor augmentation—may be more common in women with inductions and unfavorable cervices, and affect uterine contractility in the immediate postpartum period. In our study, the great majority of standard indicated inductions were performed for post-term deliveries. This raises the issue of the causal implication of this condition in the development of subsequent PPH, although there is no clear physiological hypothesis supporting the existence of such a direct impact. The independent role of a late gestational age at delivery on the risk of PPH could not be properly investigated here because of the rarity of other standard indications for induction; future research should focus on the role of late gestational age in the risk of PPH. Finally, we cannot exclude that a weak but significant association exists between induction for non standard indications and PPH, but that the power available was insufficient to detect it; however, this explanation seems unlikely because the numbers of cases and controls still provide an adequate power for a strength of association of 1.3 or more, and the estimates for the odds ratios were very closed to 1.

Even in low risk women, induction of labor, regardless of the method used, is associated with a higher risk of PPH than spontaneous labor. Induced women therefore require close monitoring for postpartum blood loss. However, this study has found no excess risk of PPH in those women who underwent induction of labor for non-standard indications. This raises the hypothesis that the increased risk of PPH associated with labor induction depends on the cervical status and may be limited to unfavorable obstetrical situations. Several studies [19], [22] have concluded that a randomized trial is needed to assess the impact of elective induction on maternal and fetal outcomes, compared with expectant management. Such trial should take into account the cervical status of women and have enough power to assess the risk of PPH and not only the risk of cesarean delivery.
